# Production of a Locus- and Allele-Specific Monoclonal Antibody for the Characterization of SLA-1*0401 mRNA and Protein Expression Levels in MHC-Defined Microminipigs

**DOI:** 10.1371/journal.pone.0164995

**Published:** 2016-10-19

**Authors:** Yoshie Kametani, Shino Ohshima, Asuka Miyamoto, Atsuko Shigenari, Masaki Takasu, Noriaki Imaeda, Tatsuya Matsubara, Masafumi Tanaka, Takashi Shiina, Hiroshi Kamiguchi, Ryuji Suzuki, Hitoshi Kitagawa, Jerzy K. Kulski, Noriaki Hirayama, Hidetoshi Inoko, Asako Ando

**Affiliations:** 1 Department of Molecular Life Science, Division of Basic Medical Science, Tokai University School of Medicine, Isehara, Kanagawa, Japan; 2 Institute of Advanced Biosciences, Tokai University, Kanagawa, Japan; 3 Department of Veterinary Medicine, Faculty of Applied Biological Sciences, Gifu University, Gifu, Gifu, Japan; 4 Teaching and Research Support Center, Tokai University School of Medicine, Isehara, Japan; 5 Department of Rheumatology and Clinical Immunology, Clinical Research Center for Allergy and Rheumatology, Sagamihara National Hospital, National Hospital Organization, Sagamihara, Kanagawa, Japan; 6 School of Psychiatry and Clinical Neurosciences, The University of Western Australia, Crawley WA, Australia; 7 Institute of Glycoscience, Tokai University, Hiratsuka, Kanagawa, Japan; CSIRO, AUSTRALIA

## Abstract

The class I major histocompatibility complex (MHC) presents self-developed peptides to specific T cells to induce cytotoxity against infection. The MHC proteins are encoded by multiple loci that express numerous alleles to preserve the variability of the antigen-presenting ability in each species. The mechanism regulating MHC mRNA and protein expression at each locus is difficult to analyze because of the structural and sequence similarities between alleles. In this study, we examined the correlation between the mRNA and surface protein expression of swine leukocyte antigen (SLA)-1*0401 after the stimulation of peripheral blood mononuclear cells (PBMCs) by *Staphylococcus aureus* superantigen toxic shock syndrome toxin-1 (TSST-1). We prepared a monoclonal antibody (mAb) against a domain composed of Y102, L103 and L109 in the α2 domain. The Hp-16.0 haplotype swine possess only SLA-1*0401, which has the mAb epitope, while other haplotypes possess 0 to 3 SLA classical class I loci with the mAb epitopes. When PBMCs from SLA-1*0401 homozygous pigs were stimulated, the SLA-1*0401 mRNA expression level increased until 24 hrs and decreased at 48 hrs. The kinetics of the interferon regulatory transcription factor-1 (IRF-1) mRNA level were similar to those of the SLA-1*0401 mRNA. However, the surface protein expression level continued to increase until 72 hrs. Similar results were observed in the Hp-10.0 pigs with three mAb epitopes. These results suggest that TSST-1 stimulation induced both mRNA and surface protein expression of class I SLA in the swine PBMCs differentially and that the surface protein level was sustained independently of mRNA regulation.

## Introduction

The class I major histocompatibility complex (MHC) antigens are constitutively expressed cellular membrane-bound glycoproteins that associate non-covalently with β-hamicroglobulin (β2M) to present intracellularly processed peptide antigens to T-cell receptors of specific CD8+ T cells [1–3]. MHC class I proteins are encoded by polymorphic genes at multiple loci, and they also act as ligands for killer-cell immunoglobulin-like receptors (KIRs) [4–6]. This polymorphism results in numerous alleles in a population, presumably to preserve the variability of the antigen presenting ability and help the species to defend against various infectious agents, although MHC variability may also cause autoimmune responses [7–9]. The main function of the classical class I MHC is the activation of cytotoxic T (Tc) cells, whereas the loss of MHC expression induces the activation of natural killer (NK) cells. In contrast, the down-regulation of classical HLA-A and HLA-B expression and up-regulation of non-classical HLA expression, such as HLA-G, negatively regulates the system of MHC-mediated immunity [10–12]. Therefore, it is important to distinguish between the classical and non-classical HLA alleles and their regulation at the level of expressed mRNAs and allele-specific surface proteins, as these different classes of MHC molecules have contrary functions. However, there are relatively few studies on the surface expression of MHC alleles, probably because of the lack of allele-specific monoclonal antibodies due to the similarity of the alleles among the MHC sequences.

The pig is an important animal model for the study of MHC function in response to infections, transplantation, and autoimmune disease [13–16]. Although the MHC molecules are known to be important for controlling infections, research on the regulation of the expression of the pig MHC genomic region, defined in pigs as the Swine Leukocyte Antigen (SLA) region, has received little or no attention to date. Most pigs have three classical SLA class I loci distributed within their MHC genomic region, and more than 100 classical SLA class I alleles have been identified [17–20]. We deduced the haplotypes in two types of mini-pig, Clawn and microminipig, and in the larger Duroc pig [21–23]. The SLA class I allele, SLA-1*0401, is one of the most frequently found alleles in various swine breeds such as Microminipig, Clawn, NIH, Yucatan, Mexican hairless mini-pigs and Meishan pigs [19, 20]. Moreover, the three-dimensional structure of this allele in complex with peptides derived from 2009-pandemic H1N1 swine-origin influenza A virus and Ebola virus has been determined in crystallographic studies [24]. Therefore, we decided to prepare a specific domain-recognizing monoclonal antibody against SLA-1*0401 and analyze its specificity using the peripheral blood mononuclear cells (PBMCs) of SLA homozygous pigs.

Swine are known to be a reservoir for methicillin-resistant *Staphylococcus aureus* (MRSA) [25–30]. Superantigens secreted by *Staphylococcus aureus* are one set of virulence factors that can induce the T cell hyper-immune response and MHC gene expression. The induction of a systemic cytokine storm by superantigens is known to create life-threatening symptoms, such as toxic-shock syndrome in newborn babies [31]. Toxic shock syndrome toxin-1 (TSST-1) is an enterotoxin of *Staphylococcus aureus* and one of the superantigens that is used to activate antigen-specific T cell clones and polyclonal T cells irrespective of the peptide presented by MHC [32, 33]. The TSST-1-reacting T cell receptor (TCR) Vβ induces a large amount of cytokine secretion containing interferon-γ (IFN-γ)Fto induce the cytotoxicity of T cells [34]. We previously reported that TSST-1 enhanced locus-specific SLA mRNA expression [35]. However, the locus-specific expression of surface SLA protein could not be detected because the only monoclonal antibodies available for the study were anti-HLA antibodies, and although they crossreact with SLA, they cannot distinguish between the different SLA loci and/or alleles [36].

Therefore, the purpose of the present study was (1) to produce and characterize a mAb that specifically recognizes SLA-1*0401, and (2) to distinguish the protein and mRNA expression levels of SLA-1*0401 using the new mAb in TSST-1 in n TSST-1 PBMCs from Microminipigs.

## Materials and Methods

### Animals and tissues

Experiments using mice for monoclonal antibody production were approved by the Institutional Animal Care and Use Committee at Tokai University and performed at Tokai University following the University guidelines. Eight-week-old female BALB/c mice were purchased from CLEA Japan (Tokyo, Japan) and maintained under specific pathogen-free conditions. Peripheral blood from adult Microminipigs was provided by Fuji Micra Inc (Fujinomiya, Japan). The blood of healthy human donors was collected at the Tokai University Hospital with informed consent, and marmoset blood was purchased from CLEA Japan. The animal health check was performed once a week. No swine or mice became ill or died prior to the experimental endpoint. For blood collection, the mice were either euthanized by inhalation of 4 to 5% isoflurane or sacrificed by cervical dislocation. Since the swine were used only for blood collection (<50 ml), no euthanasia was applied before or after bleeding them with a fine-point needle and syringe.

### Genotyping

The peripheral blood samples from Microminipigs with eight SLA haplotypes previously assigned by nucleotide sequence determination of RT-PCR products and low-resolution SLA genotyping using sequence-specific PCR primers (SSP) for the three SLA classical class I genes, SLA-1, SLA-2 and SLA-3 [21], were used in this study. Another twenty Microminipigs were genotyped for their SLA class I alleles using SSP [37], and their SLA class I haplotypes were deduced from their class I alleles and parental class I haplotypes. Of the genotyped alleles, SLA-1*0501 and SLA-1*1104 were used to predict the tertiary structure of the epitope, and SLA-1*0401 was used for the immunization of mice.

### Transfection

The transfected cDNA sequence of the swine SLA-1*0401 gene was derived from SLA-defined Clawn and Yucatan mini-pigs and based on the Sus scrofa SLA-1*0401 mRNA sequence (AB185317, AB847434, AF464002). The primers used to generate the cDNA sequences are shown in Table 1A (SLA1 N53: SLA1 C35: Table 1A). RNA was extracted from cells using TRIzol (Invitrogen, Carlsbad, CA, USA) and reverse-transcribed to cDNA using the High Capacity cDNA Reverse Transcription Kit (Life Technologies, Carlsbad, CA, USA). The sequence of the *ß2-microglobulin (ß 2M)* gene (Accession number: L13854) in GenBank was used for the design of primer sequences for *ß2M* cDNA synthesis. The primer sequences for preparation of SLA-1, SLA-2, SLA-3, SLA-6 and ß2M transfectants are summarized in Table 1A. The modified S/MAR (scaffold/matrix attachment region) episomal vectors [38] expressing SLA and β2M were used for the transfection.

**Table 1:** Primer sequences.

Locus	Allele	Primer name	Primer sequence (5' to 3')	Annealing temp. (°C)	Length of PCR products (bp)	Reference
(A) For preparation of SLA-1, SLA-2, SLA-3, SLA-6 and β2M transfectants						
*SLA-1*	SLA-1*0401	1066: SLA1 N53	TAGATATCGGTCTCATATGGGGCCTGGAGCCCTCTT	60	1,110	This study
		1067: SLA1 C35	TAGGATCCTCACACTCTAGGGTCCTTGGTAAGGGACACATCGGA			This study
*SLA-2*	SLA-2*0901	2174: SLA2 N53	TAGAATTCGAAGACAACCGGTCGCCACCATGCGGGTCAGGGGCCCTCAAGCCATC	60	1,144	This study
		2175: SLA2 C35	ATGATATCGAAGACTGGATCCTCACACTCTAGGATCCTTGGTAAG			This study
*SLA-3*	SLA-3*0602	2176: SLA3 N53	TAGAATTCGAAGACAACCGGTCGCCACCATGGGGCCTGGAGCCCTCTTCTTG	60	1,135	This study
		2177: SLA3 C35	ATGATATCGAAGACTGGATCCTCACACTCTAGGATCCTTGGTGAGAG			This study
*SLA-6*	SLA-6*0101	1068: SLA6 N53	TAGATATCGGTCTCATATGCAGGTCACGGAGCCTCG	60	1,134	This study
		1071: SLA6 C35	TAGGATCCTCAGCTTGCAGCCTGAACATAGT			This study
*β2M*		1072: β2M PIG N53	TAGATATCGGTCTCATATGGCTCCCCTCGTGGCCTT	60	380	This study
		1073: β2M PIG C35	AGGATCCTTAGTGATCTCGATCCCACTTAACTATCTT			This study
(B) For real-time PCR assay						
*SLA-1*	SLA-1*0501	SLA-1-051F	GACTCCCGCTTCATCGCCGT	60	160	This study
		SLA-1-052R	GTGAGGTGTCCCTTTGTTTCC			This study
	SLA-1*1104	SLA-1-114F	AGCCCCGTTTCATCGAAGTC	60	156	This study
		SLA-1-115R	CGCTGCCCATGACACGCCG			This study
*IFNγ*		IFN-γ-4F	GCTCTGGGAAACTGAATGACTT	53	199	Kametani et al. 2012
		IFN-γ-4R	TATTGCAGGCAGGATGACAA			Kametani et al. 2012
*IRF1*		21 1114–1134 59 52	GATCTGAAGAAGAACGTGGACACC	50	65	Kametani et al. 2012
		18 1161–1178 60 61	ATGGAGGGCAGCCTGACT			Kametani et al. 2012
*G3PDH*		G3PDH-F	GGACCTGACCTGCCGTCTG	60	450	Kametani et al. 2012
		G3PDH-R	TCCACCACCCTGTTGCTGTA			Kametani et al. 2012
(C) For sequence-based typing (SBT) method						
*SLA-1*		SLA1-9/1,2R	CCTCTTCCTGCTGCTGTCG	65	585	Ando et al. 2003
			AGCGTGTCCTTCCCCATCT			Ando et al. 2003
		SLA1-15/1,2R	GGAGCCCTCTTCCTGCTGC	61	590	Ando et al. 2014
			AGCGTGTCCTTCCCCATCT			Ando et al. 2014
		SLA1-18/1,2R	TTCCTGCTGCTGTCGGGGGC	61	581	Ando et al. 2014
			AGCGTGTCCTTCCCCATCT			Ando et al. 2003
		SLA1-3#92/r#119	CCAGACCCCGAGGCTGAGGAT	61	1,512	Ho et al. 2006
			TTCTCAATCCTTCCATTTATTTCCTC			Ho et al. 2006
*SLA-2*		SLA2-3/1,2R	GCCATCCTCATTCTGCTGTC	65	587	Ando et al. 2003
			AGCGTGTCCTTCCCCATCT			Ando et al. 2003
		SLA2-8/1.2GR	CCCTCAAGCCATCCTCATTCTG	63	596	Ando et al. 2014
			GCAGCGTGTCCTTCCCCATC			Ando et al. 2014
		SLA2-f#55/r#56R	CCACAGAATCTCCGCAGATTC	60	1,232	Ho et al. 2006
			CCGACACAGACACATTCAAATGCT			Ho et al. 2006
*SLA-3*		SLA3-5/3-6R	CCCGAGCCCTCTTCTTGCT	65	566	Ando et al. 2003
			TTTCTGGAGCCACACCACA			Ando et al. 2003
		SLA3-8/3-9R	CCGAGCCCTCTTCCTGCTG	66	565	Ando et al. 2003
			TTTCTGGAGCCACTCCACA			Ando et al. 2003
		SLA3-10/3-11R	GACCCTGGCCCTGACTGGT	68	533	Ando et al. 2003
			GGAGCCACTCCACACACGC			Ando et al. 2003
		SLA3-f#923/r#121	CCAGACTCCGAGGCTGAGGAT	61	1,494	Ho et al. 2006
			TAGGCTCTTTTCCCTTGGTTAGG			Ho et al. 2006

HEK293 cells, a cell line derived from human embryonic kidney cells, were cultured in D-MEM (GE Healthcare, Buckingham, UK)-10% FCS (Sigma-Aldrich, Co. St. Louis, MO, USA) medium and A20 cells, a BALB/c B cell lymphoma line derived from a spontaneous reticulum cell neoplasm, were cultured in RPMI 1640 (Nissui Pharmaceutical co. Ltd. Tokyo, Japan)-10% FCS medium. The cells were transfected with the cDNA sequence inserted in the N576 expression vector using the Invitrogen Neon transfection system (HEK293 cells: 1100 V, 10 ms 3 pulses; A20 cells: 1500 V, 10 ms, 3 pulses) and cultured in 5% CO_2_ at 37°C for 18 ~24 hrs. Cultured cells were collected and the expression of the mVenus reporter gene was measured by flow cytometry (Becton Dickinson, NJ, USA).

### Preparation of the peripheral blood mononuclear cells (PBMCs)

Swine peripheral blood samples were collected into a heparinized tube and centrifuged on Lymphoprep (Axis-Shield, Oslo, Norway) at 670 x g for 30 min. PBMCs were collected and washed with 10 ml of 1% (w/v) bovine serum albumin (BSA)-containing phosphate-buffered saline (PBS). The blood cells were collected by centrifuging at 350 x g for 5 min, and the remaining erythrocytes were lysed osmotically. The white blood cells were washed with PBS and used for further experiments. Human PBMC samples were purified using Ficoll Paque (GE Healthcare UK Ltd.), and mouse and marmoset PBMCs were purified using Lymphocepal.

### Monoclonal antibody preparation

We initially immunized BALB/c mice with mitomycin C (MMC, Kyowahakko-Kirin, Tokyo Japan)-treated swine PBMCs (2.2×10^6^ cells/animal). For booster treatments, MMC-treated A20 transfectants were used biweekly for 7 times with 6.8×10^5^ cells/animal at each immunization. MMC (final, 0.04 mg/ml) was added to the culture and incubated at 37°C for 30 min in 5% CO_2_. The mice were anesthetized with 20% isoflurane, and the blood was collected from the orbit. The serum antibody titers of immunized mice were checked by flow cytometry analyses using SLA-1/β2M cDNA-transfected HEK293 cells as a source of antigen. After 4 days of the final boost, mice were anesthetized with 20% isoflurane and sacrificed by blood removal; subsequently, splenocytes were fused with the mouse myeloma cell line P3-X63-Ag8-U1 according to a standard procedure [39]. Positive clones were identified by flow cytometry or by using an Imaging Analyzer (Array Scan, Thermo scientific, MA, USA). Briefly, SLA-1/β2M transfected HEK293 cells were plated in the wells of 96-well plates. Culture supernatants were added to each well, incubated for 15 min and washed twice. APC-labeled (APC: allophycocyanin) anti-mouse IgG polyclonal antibody (Poly4053; Bio Legend, San Diego, US) was added and incubated for 15 min. Plates were washed and stained with Hoechst dye (Invitrogen, Oregon, USA) for 30 min at room temperature and analyzed using the Imaging Analyzer. Positive cells were picked according to the fluorescence intensity of APC and the co-expressed mVenus fluorescent protein. The positive clones were then isolated, expanded and stocked. The mAb isotype was determined using a mouse monoclonal antibody isotyping kit (Iso Strip, Roche, Basel Schweiz).

### Flow cytometry

Cells were incubated with appropriately diluted, fluorescently-labeled primary mAb for 15 min at 4°C and washed with 1% (w/v) BSA-containing PBS. In some cases, cells were re-incubated with labeled secondary antibody. The mAbs used were as follows: anti-class-I major histocompatibility antigen (Monoclonal Antibody Center Co. Ltd, clone #PT85A), goat anti-mouse IgG1-RPE (Southern Biotech, Uden, the Netherlands). Stained cells were analyzed using the FACSVerse system (Becton Dickinson, New Jersey, USA) and FlowJo software (Tomy biochemical, Tokyo, Japan). The culture supernatant of X2F6 was also used for the primary antibody.

### Primary sequence and structure analysis of the X2F6 mAb and model building of the 3D structure

Total RNA was extracted from specific hybridoma X2F6, and the cDNA was checked for the amplification of immunoglobulin heavy- and light-chain-specific genes. Sequence reactions were performed with a GenomeLab DTCS Quick Start Kit (Beckman Coulter) and analyzed using a CEQ8000 Genetic Analysis System (Beckman Coulter). The software Genetyx was used for sequence prediction. The 3D model of the Fab fragment of X2F6 was constructed using protocols for antibody homology modeling [40] implemented in a software system, Molecular Operating Environment (MOE) (http://www.chemcomp.com, last accessed April 2014). The Protein Data Bank [41] ID code (PDB ID) of the template structure used for modeling is 3V7A [42].

### Model-building of the SLA-1 3D structure

The protein structures of SLA-1*0501 and SLA-1*1104 were constructed by the use of homology modeling protocols implemented in MOE. The X-ray crystal structure of SLA-1*0401 (PDB ID: 3QQ4) [24] was used as the template structure and the bound antigenic peptide was used to construct the 3D structures of SLA-1*0501 and SLA-1*1104.

### Stimulation of PBMCs

Swine peripheral blood samples were collected into a heparinized tube and centrifuged on Lymphocepal (IBL Co. Fujioka, Japan) at 670 x g for 30 min. PBMCs were collected and washed with 10 ml of 1% (w/v) BSA-containing PBS (PBSA) by centrifuging at 350 x g for 5 min and the remaining erythrocytes were lysed osmotically. The PBMCs were washed and cultured (2.4x10^6^/well) in RPMI 1640 medium containing 10% FCS in the presence of the toxic shock syndrome-1 (TSST-1) enterotoxin, (Toxin Tec. Sarasota, USA) at 1 μin τ ορ IΦN-γ (ITSI-Bioscience, PA, USA) at 1 ng/ml for up to 72 h at 37°C, 5% CO_2_. After 24, 48 and 72 hrs the cells were collected, washed with PBSA and used for the analyses by flow cytometry and quantitative real-time PCR.

### RNA extraction and quantitative real-time PCR

RNA was extracted from PBMCs with TRIzol (Invitrogen, Carlsbad, USA) according to manufacturer’s instructions. Total RNA concentration was determined by measuring the absorbance at 260 nm and 280 nm. The purity was estimated by the relative absorbance at 260 nm/280 nm. Integrity was assayed by agarose gel electrophoresis. The purity and integrity were greater than 95%. cDNA was synthesized from the total RNA (2 μtha σψντηa High Capacity cDNA Reverse Transcription Kit (Applied Biosystems, CA, USA).

Three sets of previously published specific primers against the swine genes *IFN-γ*, *IRF-1* and the housekeeping gene *glyceraldehyde 3-phosphate dehydrogenase* (*GAPDH*) were used to determine the time course of gene expression levels in PBMCs of Microminipigs by real-time PCR [35]; Table 1). Another two sets of SLA-1*0401 and SLA-1*1104 allele-specific primers were designed to amplify SLA-1*0401 and SLA-1*1104 alleles mRNA (Table 1). The *GAPDH*-specific primer set was used as an internal control for the other three genes. The expression levels of the two *SLA-1* alleles, *IFN*-*γ*, *IRF-1* and *GAPDH* were measured by real-time PCR using an ABI PRISM 7500 Fast Sequence Detector System (Applied Biosystems, CA) with Fast SYBR^®^ Green Master Mix (Applied Biosystems, CA, USA). The synthesized cDNAs were used as templates and were amplified using the allele specific primer sets of *SLA* and *IFN-γ*, *IRF-1* and *GAPDH*. The 10 μl amplification reaction volume contained 50 ng of cDNA, 0.5 units of high fidelity Gold *Taq* polymerase (Applied Biosystems, CA, USA), 10 x PCR buffer, 2.5 mM MgCl_2_, 2 mM of each dNTP and 0.5 mM of each primer. The cycling parameters were as follows: 25 cycles of 98°C/10 sec, 62°C/30 sec and 68°C/30 sec. Melting curve analysis showed that there was no primer dimer formation. The relative quantitative values were calculated by the comparative C(T) method, also referred to as the 2(-DeltaDeltaC(T)) method [43, 44]. Serial dilutions of cDNA were amplified by real-time PCR using gene-specific primers. A plot of log cDNA dilution versus delta threshold cycle (Ct) value gives an absolute value of the slope. The absolute value of the slope (z) was calculated using an approximate formula, y = -3.32x+z. The 2-<DELTA><DELTA>Ct value is given by [(Ct target gene—Ct internal control) Time X—(Ct target gene—Ct internal control) Time 0]]. Each Ct value was determined in the optimal cDNA dilution condition with the range of 100%±5% PCR efficiency.

### Statistical analysis

Results were presented as the means ± SE. Data from the lymphocyte activation assay and real-time PCR were analyzed using Student’s *t*-test to determine the significance of the treatment. In all statistical analyses, a *P* value of <0.05 was considered significant.

## Results

### Preparation of the monoclonal antibody X2F6

Swine SLA-1*0401 and *β2 microglobulin* cDNAs were inserted into N576, an expression vector containing a monomer Venus yellow—green fluorescent protein (mVenus)-reporter gene, and the modified vector was transfected into A20 cells. The expression of the mVenus gene was observed in more than 50% of the transfected cells at 18 to 24 hrs after transfection. BALB/c mice were injected with mitomycin C (MMC)-treated swine PBMCs and then with MMC-treated N576-A20 cells as the booster immunizations. The titer of the antiserum against the SLA-1*0401 expressed by the N576-HEK293 cells was increased successfully after the second booster. The crossreactivity between N576-HEK293 and the antisera was confirmed by flow cytometry, and the mice with sera that achieved a peak mean fluorescence intensity (MFI) of more than 30 were selected and used for monoclonal antibody preparation.

**Fig 1:**
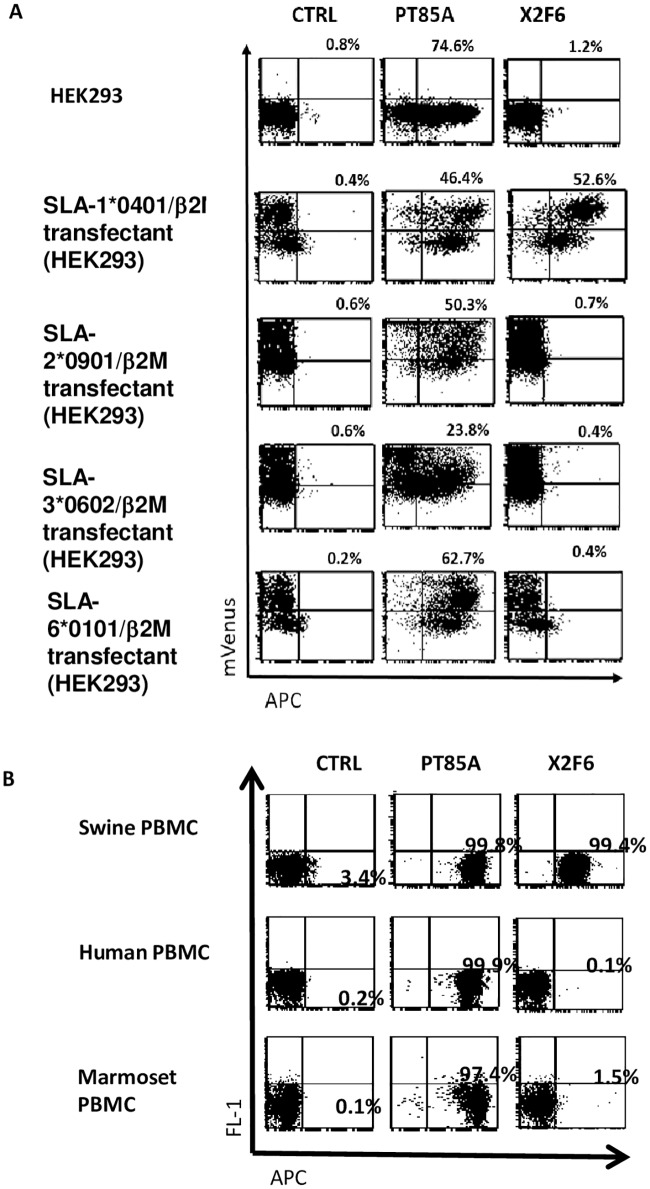
Specificity of the X2F6 mAb. (A) SLA-1*0401, SLA-2*0901, and SLA-3*0602, which are the classical class-I SLA alleles of Haplotype Hp-16.0, and SLA-6*0101, which is a non-classical class-I SLA allele of Hp-16.0, were transfected into HEK293 parent cells, and the reactivity of X2F6 (right panels) was examined by flow cytometry (FCM). Propidium iodide (PI) positive-dead cells were avoided for the gating. PT-85A, the pan-specific MHC class-1 antibody, was used for the positive control (middle panels). (B) The species specificity was examined using swine (Hp-16.0), human, and common marmoset PBMCs. Lymphoid gate was used for the analysis. The percentages shown above the panels are the double-positive cell percentages.

We selected a specific X2F6 subclone (Fig 1 and S1 Fig) for further use because this clone secreted a monoclonal antibody (mAb) that reacted with the HEK293 transfectants expressing SLA-1*0401 (with an MFI greater than 3x10e3) and the *mVenus* fluorescent protein with similar or identical staining patterns. All of the X2F6 subclones showed similar staining patterns, suggesting that the cross-reacting proteins were stable expression products. The staining pattern was compared with that of PT-85A, a commercial pan-specific mAb against MHC. PT-85A stained not only classical class I SLA but also SLA-6, a non-classical class I SLA. The pattern of SLA-1-transfectant staining by the selected X2F6 clone was similar to that seen with the commercial PT-85A clones (Fig 1A). The isotype of this mAb was IgG2aκ.

### Characterization of X2F6 specificity

We checked the species specificity of the X2F6 mAb using swine, human and common marmoset PBMCs. X2F6 recognized only the swine PBMCs, and no reactivity was observed with the human and common marmoset PBMCs, suggesting that the mAb was swine specific (Fig 1B). Next, we examined the locus specificity of the mAb using classical class I SLA, SLA-2*0901, and SLA-3*0602 and non-classical class-I SLA, and SLA-6*0101, which are contained in the same haplotype (Hp-16.0) as the SLA-1*0401 antigen, using transfectants expressing each of these genes. The flow cytometry analysis revealed that SLA-2*0901, SLA-3*0602 and SLA-6*0101 transfectants were not recognized by X2F6, whereas the SLA-1*0401 transfectant was recognized by X2F6 (Fig 1A). These results suggest that X2F6 is specific for the SLA-1 locus in Hp-16.0.

**Fig 2:**
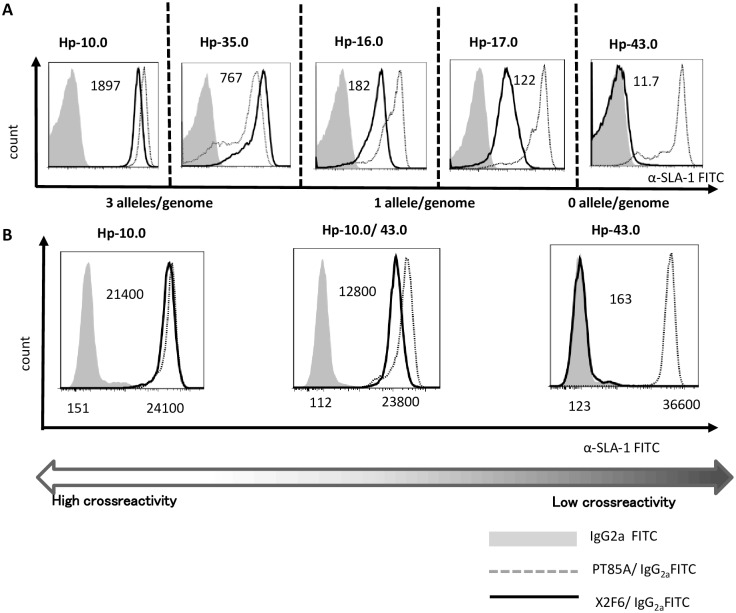
Haplotype-specific reactivity of the X2F6 mAb. (A) Microminipig PBMCs of each haplotype were stained with X2F6 or PT85A followed by anti-mouse IgG-FITC and analyzed by FCM. Lymphoid gate was used for the analysis. Solid lines represent the X2F6-stained patterns. Broken lines represent PT85A-stained patterns. Filled lines represent the isotype-control-stained patterns. MFI scores for each haplotype are shown in the panels. The YLL type-allele number is also shown. (B) The MFIs of Hp-10.0 and Hp-43.0 haplotypes were selected and the homozygous pig PBMCs and heterozygous pig PBMCs were compared. The upper panels are the data from FACSCalibur, and the MFI score is significantly lower than in the lower panels, whereas the relative levels of the surface protein are comparable.

To examine the allele specificity of X2F6 for haplotypes other than Hp-16.0, we tested the reactivity of X2F6 against the PBMCs of four other swine homozygous haplotypes that are shown in Table 2. The swine PBMCs collected from each of the duplicate homozygous haplotypes were stained with X2F6 or the pan-specific anti-class-I MHC mAb PT85A followed by fluorescently-labeled anti-mouse IgG and analyzed by flow cytometry. All five haplotypes showed different staining intensities (Fig 2A). The X2F6 antibody reacted with Hp-10.0 to produce the highest MFI, whereas Hp-35.0 showed the second highest MFI. The haplotypes Hp-16.0, which contained the immunized antigen SLA-1*0401, and Hp-17.0 both had an intermediate MFI. No reactivity was observed for Hp-43.0 (Table 2). On the other hand, the Hp-43.0 and Hp-10.0 heterozygous pigs reacted with X2F6 to produce an intermediate intensity that was lower than that in the Hp-10.0 homozygous pigs and higher than that in the Hp-43.0 homozygous pigs (Fig 2B). These results showed that X2F6 was specific to swine PBMCs and that it recognized different classical class I SLA alleles at different loci in a haplotypic manner.

**Table 2:** SLA class I genotypes and haplotypes deduced from SBT and sequence-specific primer (SSP) methods and MFI scores for Microminipigs.

Breed	Sample No.	Class I haplotype (Hp-)	class I			MFI (X2F6)^1^
			*SLA-1*	*SLA-3*	*SLA-2*	
Microminipig	320, 831, 2303, 2316, 2567,1083	10.0	*0501	*0801	*0302	1897
	807, 982	35.0	*1201, *1301	*0502	*1001	767
	965, 1938	16.0	*0401	*0602	*0901	182
	1003	17.0	*0804	*0305	*0603	122
	1173, 1495, 2024, 2030, 2259	43.0	*1104	*0401	*040202	11.7
	1599, 1810, 1932	10.0/43.0	*0501, *1104	*0801, *0401	*0302, *040202	—

^1^MFI scores for PBMCs of Microminipigs with each of the SLA class I haplotypes that were reacted with X2F6 Mab.

### Sequence analysis and SLA epitope recognition of X2F6

**Fig 3:**
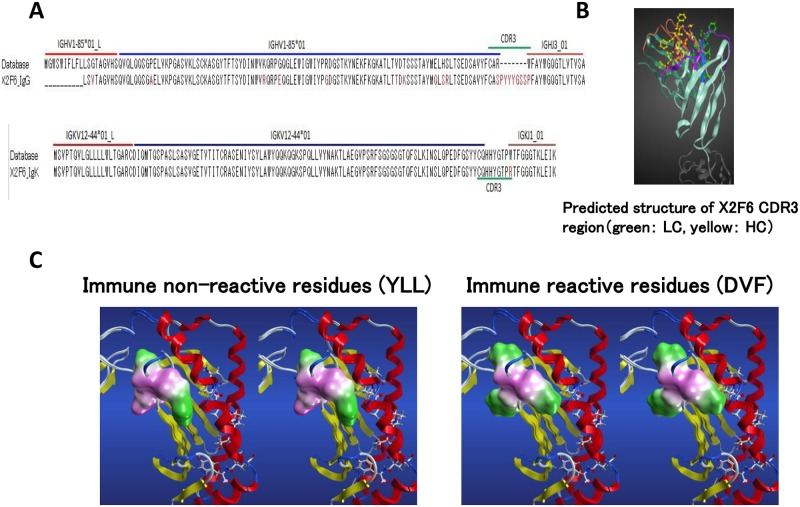
Tertiary structure of X2F6 mAb and the predicted antibody epitope. (A) Amino acid sequences of heavy and light chains of the X2F6 variable region. The database sequence PDB ID 3V7A is shown as the control sequence. (B) The predicted tertiary structure of the X2F6 mAb. (C) The tertiary structures of the YLL set in SLA-1*0501, which reacts with X2F6 with high reactivity (left panel), and the DVF set in SLA-1*1104, which cannot react with X2F6 (right panel), are shown. Pink (hydrophobic) and green (hydrophilic) colors represent the amino acid character. The structure is largely different, and the binding affinity is predicted to be different.

**Fig 4:**
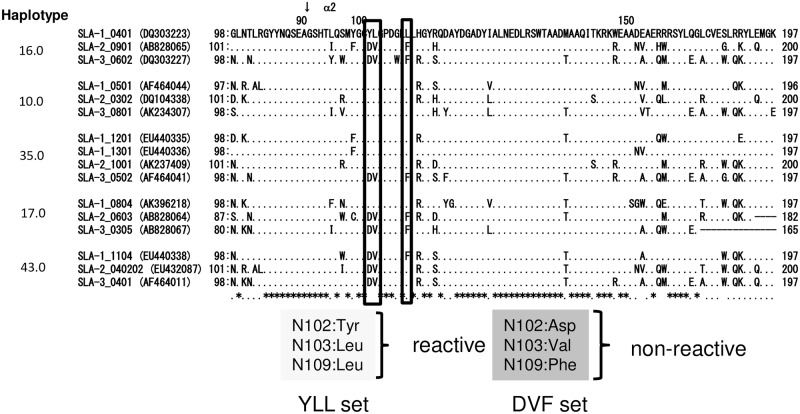
Amino acid alignment of each classical class I allele in five SLA class I haplotypes. The amino acid sequence alignment of the alleles of each SLA locus is shown. For the haplotypes with a specific set of amino acids (Y102, L103, L109; the YLL set), in which each allele reacted with X2F6, the number of YLL sets determined the level of reactivity. The MFI for X2F6 reactivity was highest in the Hp-10.0 PBMCs that possessed three YLL sets in the SLA-1, SLA-2 and SLA-3 loci.

The amino acid sequences and variable regions of the X2F6 antibody are shown in Fig 3A for both the heavy and light chains. The protein database sequence 3V7A is shown above the X2F6 sequence. The tertiary structure of X2F6 was also predicted (Fig 3B). To predict the SLA epitope recognized by X2F6, we aligned the amino acid sequences of SLA-1, SLA-2 and SLA-3 alleles for each of the five haplotypes (Fig 4). We compared the antigenic sequence of SLA-1*0401 with the non-antigenic sequences of SLA-2*0901, SLA-3*0602, Hp-43.0 SLA-1*1104, SLA-2*040202 and SLA-3*0401 that did not react with X2F6. Amino acid sequence differences were found in the α2 domain, specifically, Y102, L103, and L109 (YLL set) in SLA-1*0401 compared to D102, V103, and F109 (DVF set) in the other alleles. Hp-10.0 and Hp-35.0, which reacted with X2F6, also had alleles with the YLL set. Interestingly, the allelic YLL set number and MFI exhibited strict correlation (Fig 2). The predicted 3D structures of SLA-1*0501 and SLA-1*1104 clearly show that the amino acids of the YLL and DVF sets are exposed and clustered near a loop region on the surface of these SLA molecules, respectively. As shown in Fig 3C, the structures and the surface characters of the YLL and DVF sets are significantly different.

These results predict that the antibody X2F6 will only recognize the SLA region composed of Y102, L103 and L109 residues. Thus, in the case of the Hp-16.0 haplotype, the antibody will only recognize the SLA-1 locus and the antigenic allele SLA-1*0401 that contains the YLL epitope.

### Correlation of the mRNA and surface protein expression of SLA

To analyze the surface protein level of the SLA allele, we used X2F6 and homozygous Microminipigs with Hp-16.0 (n = 2) to detect the SLA-1*0401 protein level on the TSST-1-stimulated PBMC surface. The SLA-2 and SLA-3 alleles of Hp-16.0 have the DVF set, D102, V103, and F109, in their α2 domains, as mentioned above. These domains are predicted not to react with X2F6 (Fig 4). We also examined Hp-43.0, whose classical class-I SLA alleles have the DVF and not the YLL set. Both SLA-1*0401 and SLA-1*1104 mRNA were analyzed by real-time-PCR after stimulation.

**Fig 5:**
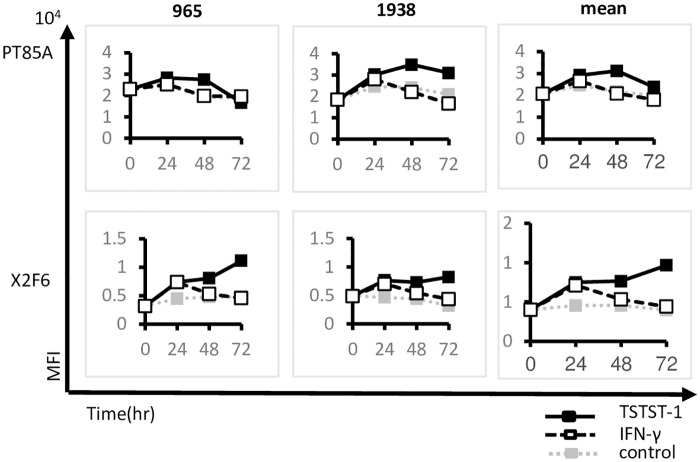
The quantification of the SLA-1*0401 protein after TSST-1 stimulation. The PBMCs of two pigs with the Hp-16.0 haplotype (individuals #965 and #1938) were examined for the surface protein level of SLA after the stimulation. X2F6 mAb was used to determine the SLA-1*0401 surface protein level (upper panels). PT-85A was used for the positive control (lower panels). Closed squares with a solid line show TSST-1-stimulated PBMCs, open squares with a broken line show IFN-γ stimulation. Closed squares with a dotted line show the negative control.

First, PBMCs from each haplotype were stimulated with TSST-1 or IFN-γ, Fand the surface expression of SLA was analyzed serially. IFN-γ or TSST-1 stimulation enhanced the surface protein expression of SLA-1 in the PBMCs (Fig 5). However, while the protein expression peaked at 24 hrs and was down-regulated thereafter during IFN-γ treatment, the surface protein expression was sustained at near-maximum levels for up to 72 hrs during TSST-1 stimulation. In contrast, no cross-reactivity was detected in homozygous pigs with SLA-1*1104, irrespective of the stimulation (S2 Fig). When PT85A, a pan-specific anti-class-I MHC antibody, which crossreacts with human and swine, was used for the detection of surface SLA molecules, the peak was at 24 to 48 hrs and decreased slowly thereafter. As for Hp-10.0 (n = 3), which possesses three YLL sets, a similar complement of surface level SLA molecules was observed (S2 Fig). As for the heterozygotes of Hp-10.0 and Hp-43.0, intermediate reactivity was observed, but the kinetics were similar to those observed for Hp-10.0 homozygotes.

**Fig 6:**
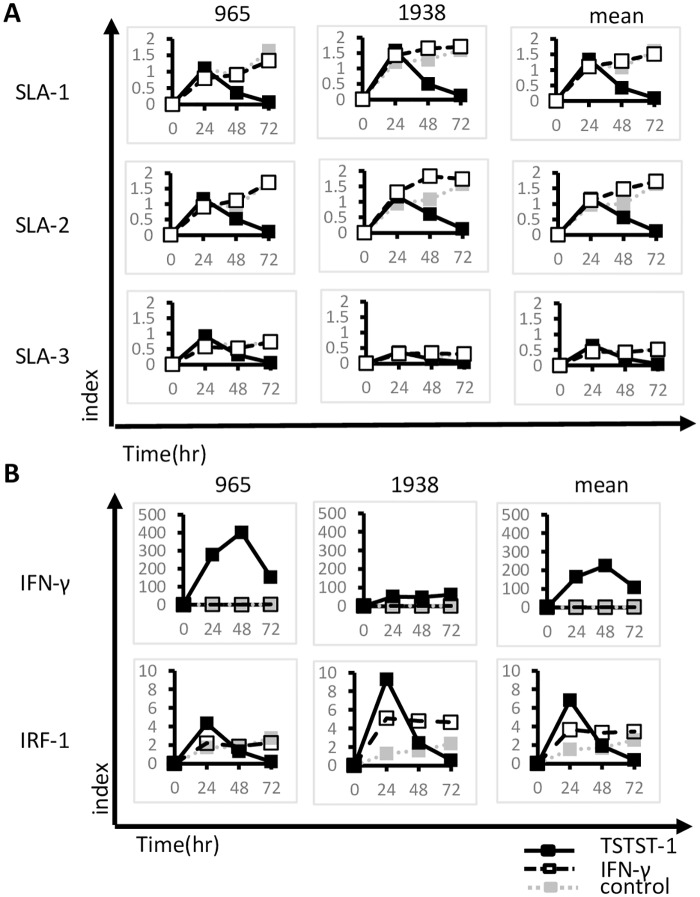
Class I SLA-related mRNA expression after TSST-1 stimulation. The PBMCs of two pigs with the Hp-16.0 haplotype (individuals #965 and #1938) were examined for classical class I SLA (A) and related mRNA (B) expression after stimulation. Closed squares with a solid line show TSST-1-stimulated PBMCs, open squares with a broken line show IFN-γ stimulation, and closed squares with a dotted line show the negative control.

We also analyzed the mRNA level by real-time PCR with allele-specific primers and found that with TSST-1 stimulation, the SLA-1^*^0401 and SLA-1*1104 mRNA expression levels peaked at 24 hrs and decreased thereafter to reach base-line levels at 72 hrs after TSST-1 treatment (Fig 6A). The mRNA expression results obtained for the IFN-γ stimulation study were diverse among individuals and did not correlate strictly with SLA-1*0401 expression (Fig 6B and data not shown). In addition, the *IRF-1* mRNA expression levels basically correlated with the SLA-1*0401 mRNA expression (Fig 6B). Additionally, the mRNA expression levels of SLA-1*0501 and SLA-1*1104 were similar to that of SLA-1*0401 (S3 Fig). However, a strict correlation between SLA-1 and IRF-1 mRNA was not observed for the alleles SLA-1*0501 and SLA-1*1104 (data not shown). Contrary to this, as observed for IFN-γ stimulation, the SLA-1*0401, SLA-1*0501 and SLA-1*1104 mRNA levels did not differ significantly, and the expression levels increased or were maintained at maximum levels from 24 hrs to 72 hrs after treatment (Fig 6A and S3 Fig). In contrast, IFN-γ stimulation did not induce IFN-γ and IRF-1 mRNA expression above the levels stimulated by TSST-1, except for the IRF-1 mRNA expression level at 72 hrs (Fig 6B).

Collectively, these results suggest that the kinetics of SLA-1*0401 allele expression were different at the mRNA and protein levels. In particular, TSST-1 stimulation induced quite different features of SLA-1 mRNA expression and surface protein expression compared to IFN-γ stimulation, although the SLA-1 mRNA level correlated with IRF-1 mRNA level.

## Discussion

As the pig is recognized to be a useful experimental animal, we genotyped and haplotyped a number of different breeds to use SLA-defined pigs for infection and transplantation studies involving SLA-related reactions [21–23]. In this regard, immunodeficient pigs [45], iPS cells from the SLA-defined minipig [46], and transplantation of human iPS cells into the pigs [47] were established to advance a breakthrough for these types of studies on middle-sized experimental animals. However, the development of tools and reagents to distinguish SLA alleles is still urgently needed to drive progress in studies of the role of polymorphisms in the regulation and function of SLA gene expression.

In this study, we prepared and characterized a specific mAb that recognized a unique amino acid cluster, YLL, in the classical class I SLA tertiary structure and not the allelic DVF cluster. This was validated using a panel of SLA transfectants, human and common marmoset PBMCs and haplotype-defined Microminipig PBMCs. We undertook a predictive analysis of the alleles of three classical class I loci, SLA-1, SLA-2 and SLA-3, that might be recognized by X2F6 based on the sequences available at the IPD-MHC SLA website and those that were submitted recently to public DNA sequence databases [http://www.ebi.ac.uk/ipd/mhc/sla/index.html, 19, 20]. Of the 60 known SLA-1 alleles, 43 alleles were identified to have a unique amino acid cluster, YLL, that could be expected to react with the X2F6 antibody. In contrast, only 19 alleles and one allele of the 82 SLA-2 and 31 SLA-3 alleles, respectively, were found to have the unique amino acid cluster. Therefore, X2F6 might recognize most of the SLA-1 alleles and only a few of the SLA-2 and SLA-3 alleles.

In our previous study, the correlation of SLA allele mRNA and protein expression was not clear because of the lack of specificity of the mAb, PT85A. To use our new mAb, we selected a haplotype that could be used to examine the expression of a single locus and allele encoding a class I SLA protein, as the number of YLL and DVF sets in the class I SLA alleles were different among the haplotypes. This mAb possessed high specificity and recognized a specific locus and allele when we selected the haplotype Hp-16.0. Collectively, these tools enabled us to specifically analyze the kinetics of surface SLA protein expression.

Using this combination of tools, we characterized the kinetics of the surface protein levels of the SLA-1*0401 allele after stimulating the haplotyped PBMCs for 72 hrs with TSST-1 or with IFN-γ. Both stimulants enhanced the surface protein expression, but after 48 hrs of TSST-1 stimulation, the mRNA expression was decreased. In contrast, the surface protein level was maintained until 72 hrs after the stimulation. The IFN-γ mRNA level was not closely correlated with the SLA-1*0401 mRNA level, suggesting that the TSST-1 stimulation modified the expression of SLA-1 mRNA not only by inducing a large amount of translatable IFN-γ but also through other complex regulatory systems. This result regarding the differential expression of the mRNAs and proteins of different MHC alleles is not controversial and was previously observed for the HLA-A and HLA-B molecules expressed by the HEK293T cell line [48]. Another haplotype, Hp-10.0, with plural YLL sets showed similar kinetics, suggesting that the surface protein level of three classical SLA class I loci may be similarly regulated by TSST-1 stimulation.

Although the reason why the mRNA level was not directly correlated with the surface protein expression is not clear, the regulatory systems for transcription, translation and post-translation processes are complicated, and the SLA-1 molecules may be affected at various steps during protein translation or during recruitment on the cell surface [49]. Because IFN-γ is reported to play a role in the expression of the antigen-processing machinery (APM) [10], the mRNA and surface SLA protein expression is usually thought to be induced by signals downstream of the IFN-γ signal, such as Janus activating kinase 2 (JAK2) suppression. Otherwise, structural modification of Tapasin and related molecules may be induced and the peptide loading complex may change its peptide-binding affinity [50]; alternatively, the recruitment of SLA molecules by cargo proteins such as Bap31 may be changed [51]. However, our results show that IFN-γ expression is not directly correlated with the surface expression of SLA. As for the interferon stimulated response element (IRSE), which is a target of IFN-γ, the sequence is different among the loci, and for example, HLA-A is reported to not easily respond to IFN-γ [52, 53]. Therefore, we may need to consider mechanisms other than IFN-γ modification to explain the discrepancy between mRNA and surface protein expression. After activation, T cells can induce IFN-γ expression along with the closing of the interleukin 2 (IL-2) promoter, as we reported previously [54]. Such an anergic state may be induced along with MHC down-regulation in the cells. Otherwise, by the extensive internalization of the SLA molecules after active TCR signal transduction, intracellular SLA proteins might be increased and the surface protein level might be decreased. Such specific modifications of cell surface expression may be clarified using our system in future studies.

Allele-specific transcription of classical class I MHC is mainly correlated with Enhancer A, ISRE and SXY, for which allele-specific transcriptional regulation was reported [55, 56]. The SXY module contains binding motifs for activating transcription factors (ATF)/cyclic AMP response element binding protein (CREB) [57], class-II trans-activator (CIITA) [58], and nuclear factor Y (NFY) [59]. Previous studies on polymorphisms in HLA regulatory regions have demonstrated that the correlation between diseases and HLA haplotype-specific gene expression is due to 3’-UTR sequence diversity and mRNA stability. However, these reports have not considered the differences in mRNA and protein expression regulation after stimulation by TSST-1 or IFN-γ. In this regard, our experimental system may help in the future to clarify these observed differences.

In our study, the X2F6 and PT85A antibodies detected different kinetics of SLA surface expression. While X2F6 detected only the SLA-1*0401 molecule, PT85A might have detected other class I SLA molecules and non-classical molecules, such as SLA-6 (Fig 1). As non-classical class I MHC is reported to have a different regulation system from classical class I MHC in human and mouse species [60], the swine non-classical class I MHC might also have a different regulation system. The different regulation of each class I SLA might affect the kinetics of the X2F6- and PT-85A-recognized surface SLA molecules.

Superantigens such as TSST-1 may induce a cytokine storm, which is life threatening for newborns and the old [31]. If the surface protein expression of MHC class I is maintained after mRNA down-regulation, the cells may continue to stimulate CD8 T cells to secrete Th1 cytokines and attack specific target cells. Moreover, the large amount of cytokines may induce tissue damage in the host and cause sepsis. Therefore, it is critical to monitor MHC class I expression at the protein level. Moreover, if the mechanism for sustained surface protein expression is elucidated, it may help to develop molecular targeting reagents for the treatment of such deleterious cytokine storms.

In conclusion, we established a new SLA-1*0401 allele-recognizing monoclonal antibody, X2F6, and used it to find marked differences between mRNA and surface protein expression of the SLA-1*0401 allele during stimulation with TSST-1 of *staphylococcus aureus*. The X2F6 mAb and haplotype-defined Microminipigs in combination with next generation sequencing [61] may help to clarify the regulation of MHC gene expression in more detail in future studies.

## Supporting Information

S1 Fig Array Scan Images of the Specificity of the X2F6 mAb. Hybridoma screening was performed by Array Scan (Thermo Fisher co. Ltd). Upper panels show the crossreactivity of the X2F6 clone culture supernatant with either HEK293 or SLA-1/βmVenus (transfected gene expression) and APC-labeled secondary antibody (surface SLA recognition) are also shown. Lower panels show the same patterns using PT-85A, a pan-specific MHC class I antibody.
(TIF)

S2 Fig Surface expression of Class I SLA of Hp10.0. Stimulated PBMCs of Hp-10.0 homozygous pigs (n = 3) and Hp-10.0 and Hp-43.0 heterozygous pigs (n = 3) were examined for surface class I SLA protein expression after TSST-1 or IFN-γ stimulation. Closed squares with solid lines show TSST-1 stimulated PBMCs, open squares with broken lines show IFN-γ, and closed squares with dotted lines show the negative control.
(TIF)

S3 Fig mRNA Expression of the SLA-1 gene. Stimulated PBMCs of Hp-10.0 homozygous pigs (n = 3) and Hp-10.0 and Hp-43.0 heterozygous pigs (n = 3) were examined for the expression of the SLA-1*0501 and SLA-1*1104 mRNAs after TSST-1 or IFN-γ. Closed squares with solid lines show TSST-1-stimulated PBMCs, open squares with broken lines show IFN-γ, and closed squares with dotted lines show the negative control.
(TIF)
