# Supplementary figures and images for: Production of a Locus- and Allele-Specific Monoclonal Antibody for the Characterization of SLA-1*0401 mRNA and Protein Expression Levels in MHC-Defined Microminipigs
Source: PLoS One. 2016 Oct 19;11(10):e0164995. doi: 10.1371/journal.pone.0164995 (PMC5070868; doi:10.1371/journal.pone.0164995)

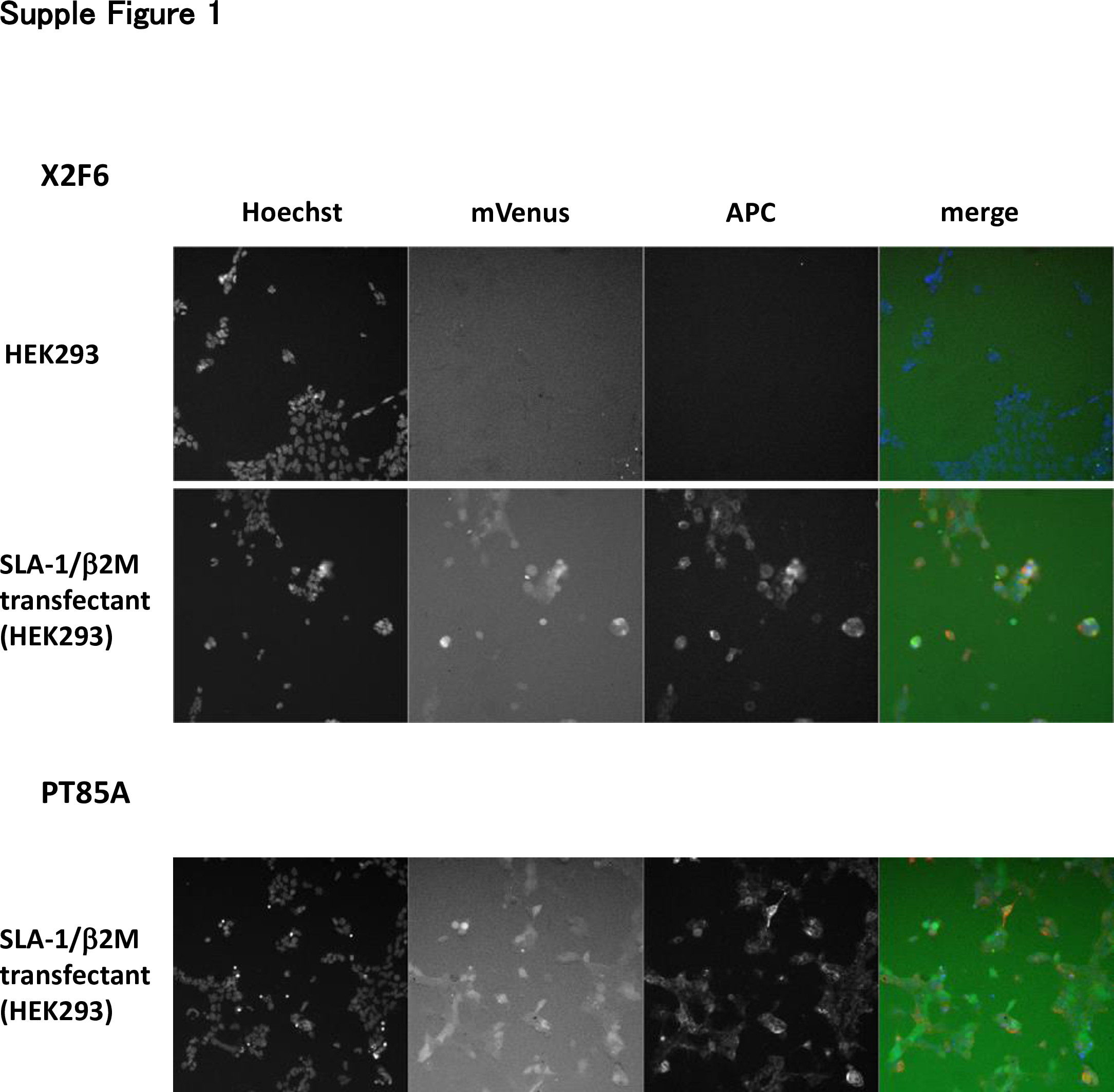

Supplement: S1 Fig — Hybridoma screening was performed by Array Scan (Thermo Fisher co. Ltd). Upper panels show the crossreactivity of the X2F6 clone culture supernatant with either HEK293 or SLA-1/βmVenus (transfected gene expression) and APC-labeled secondary antibody (surface SLA recognition) are also shown. Lower panels show the same patterns using PT-85A, a pan-specific MHC class I antibody. (TIF) [file pone.0164995.s001.tif]

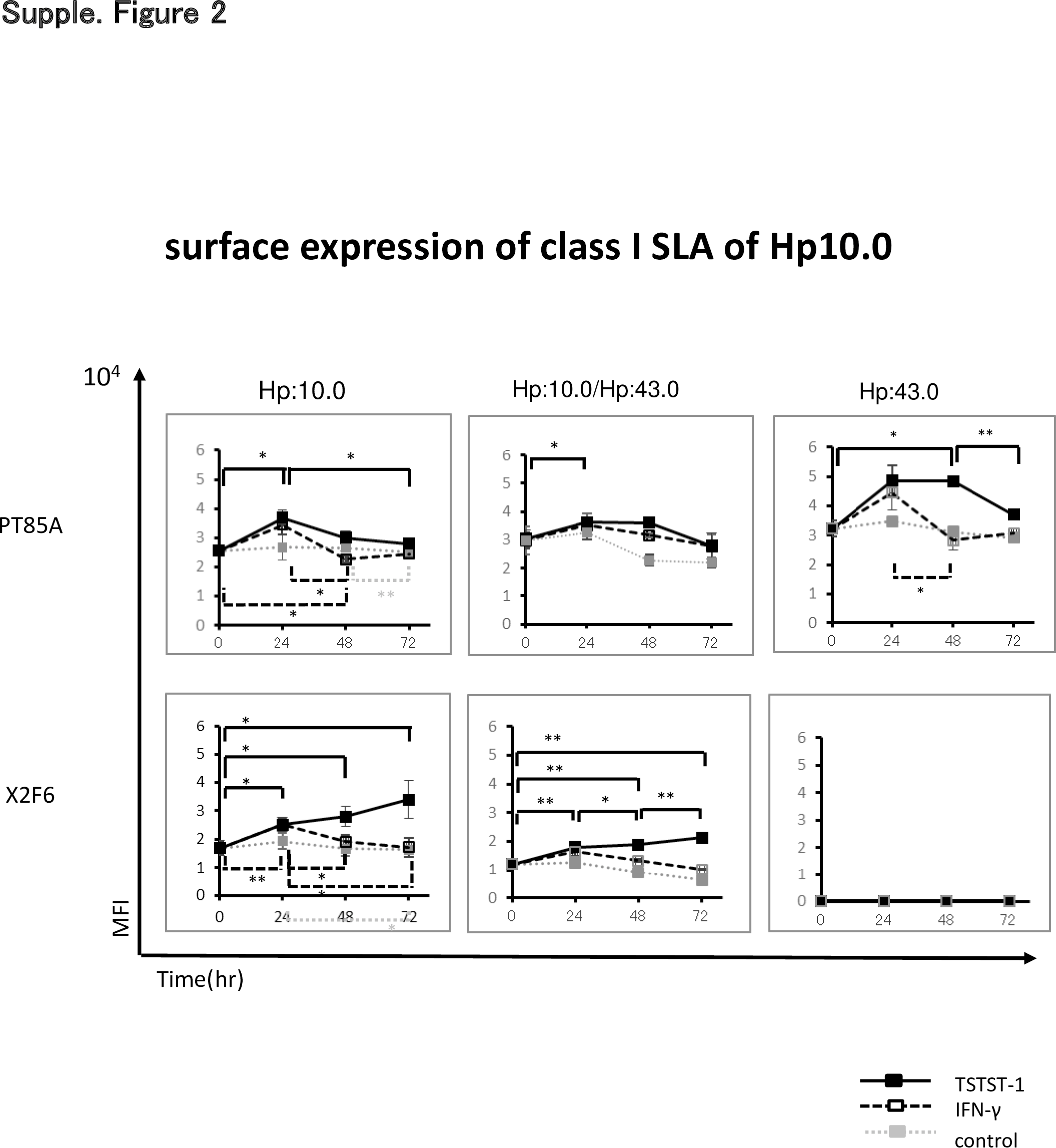

Supplement: S2 Fig — Stimulated PBMCs of Hp-10.0 homozygous pigs (n = 3) and Hp-10.0 and Hp-43.0 heterozygous pigs (n = 3) were examined for surface class I SLA protein expression after TSST-1 or IFN-γ stimulation. Closed squares with solid lines show TSST-1 stimulated PBMCs, open squares with broken lines show IFN-γ, and closed squares with dotted lines show the negative control. (TIF) [file pone.0164995.s002.tif]

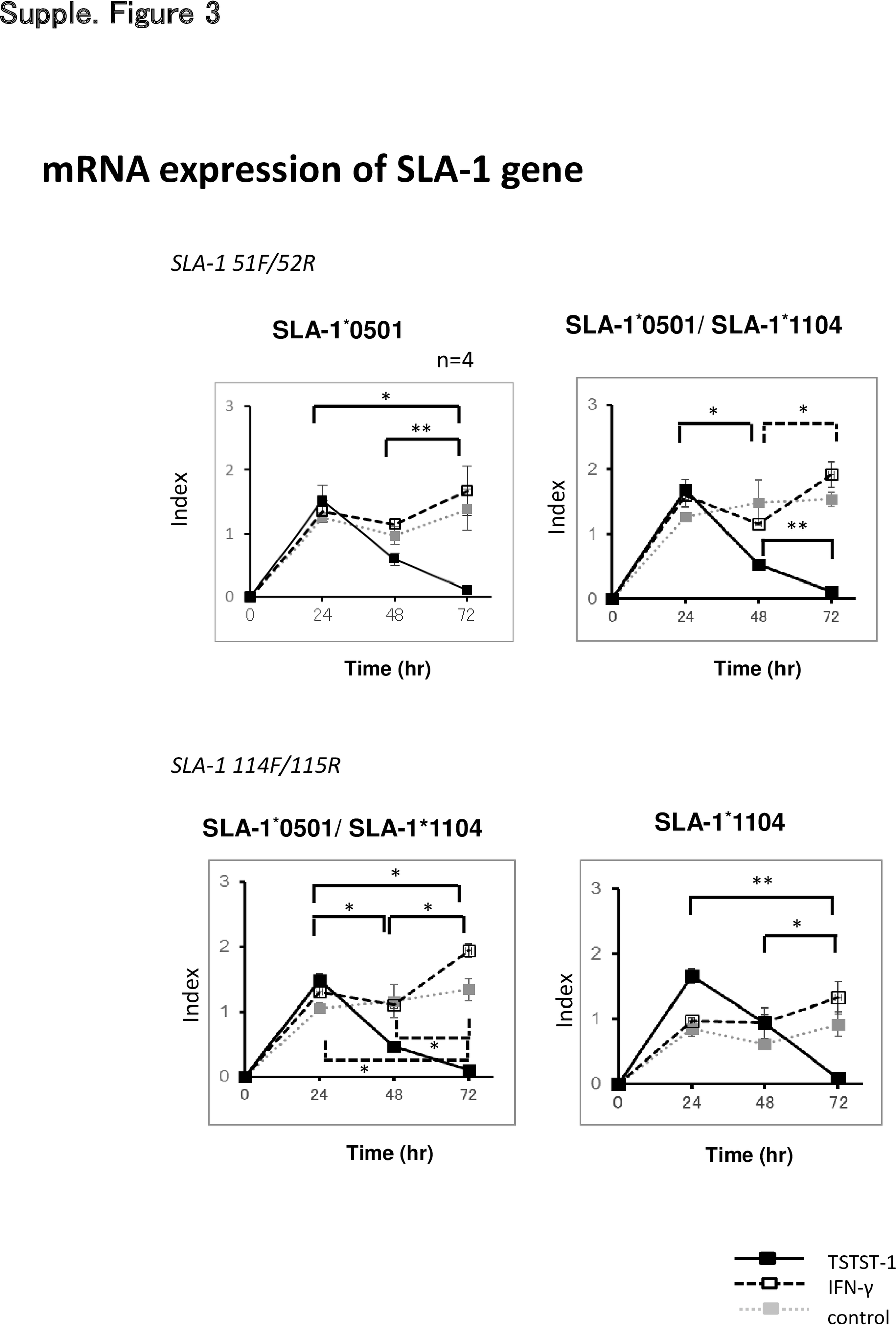

Supplement: S3 Fig — Stimulated PBMCs of Hp-10.0 homozygous pigs (n = 3) and Hp-10.0 and Hp-43.0 heterozygous pigs (n = 3) were examined for the expression of the SLA-1*0501 and SLA-1*1104 mRNAs after TSST-1 or IFN-γ. Closed squares with solid lines show TSST-1-stimulated PBMCs, open squares with broken lines show IFN-γ, and closed squares with dotted lines show the negative control. (TIF) [file pone.0164995.s003.tif]
